# Study on Bacteria Isolates and Antimicrobial Resistance in Wildlife in Sicily, Southern Italy

**DOI:** 10.3390/microorganisms9010203

**Published:** 2021-01-19

**Authors:** Delia Gambino, Domenico Vicari, Maria Vitale, Giorgia Schirò, Francesco Mira, Maria La Giglia, Alessandra Riccardi, Antonino Gentile, Susanna Giardina, Anna Carrozzo, Valentina Cumbo, Antonio Lastra, Valeria Gargano

**Affiliations:** 1 Istituto Zooprofilattico Sperimentale della Sicilia “A. Mirri”, Via Gino Marinuzzi n. 3, 90129 Palermo, Italy; deliagamb@gmail.com (D.G.); maria.vitale@izssicilia.it (M.V.); giorgia.schiro91@gmail.com (G.S.); dottoremira@gmail.com (F.M.); maria.lagiglia@izssicilia.it (M.L.G.); antogentile1980@gmail.com (A.G.); susbi@live.it (S.G.); anna.carrozzo@izssicilia.it (A.C.); valentina.cumbo@gmail.com (V.C.); antonio.lastra@izssicilia.it (A.L.); valeria.gargano@izssicilia.it (V.G.); 2 Department of Veterinary Science, University of Pisa, Viale delle Piagge n. 2, 56124 Pisa, Italy; riccardialessandra3@gmail.com

**Keywords:** wildlife, antibiotic resistance, resistance genes, *int1*, One Health

## Abstract

Wild environments and wildlife can be reservoirs of pathogens and antibiotic resistance. Various studies have reported the presence of zoonotic bacteria, resistant strains, and genetic elements that determine antibiotic resistance in wild animals, especially near urban centers or agricultural and zootechnical activities. The purpose of this study was the analysis, by cultural and molecular methods, of bacteria isolated from wild animals in Sicily, Italy, regarding their susceptibility profile to antibiotics and the presence of antibiotic resistance genes. Bacteriological analyses were conducted on 368 wild animals, leading to the isolation of 222 bacterial strains identified by biochemical tests and 16S rRNA sequencing. The most isolated species was *Escherichia coli*, followed by *Clostridium perfringens* and *Citrobacter freundii*. Antibiograms and the determination of resistance genes showed a reduced spread of bacteria carrying antibiotic resistance among wild animals in Sicily. However, since several wild animals are becoming increasingly close to residential areas, it is important to monitor their health status and to perform microbiological analyses following a One Health approach.

## 1. Introduction

Sicily is the largest island in the Mediterranean Sea and the biggest Italian region. Located in the middle of the Mediterranean area, Sicily has a strategically important position for bird migration because it represents a natural bridge between Europe and Africa. The Sicilian territory is characterized by a considerable extension of rural areas and natural parks and by the presence of urbanized and industrial areas. These regional parks, such as Parco delle Madonie and Parco dei Nebrodi, as well as several smaller regional reserves, host many wildlife mammal species, including wild boar (*Sus scrofa*), wild rabbit (*Oryctolagus cuniculus*), fox (*Vulpes vulpes*), and porcupine (*Hystrix cristata*), and many common birds together with endangered species, such as the golden eagle (*Aquila chrysaetos*), the smaller Bonelli’s eagle (*Aquila fasciata*), and the griffon vulture (*Gyps fulvus*).

Wildlife diseases represent an aspect of veterinary medicine that is still lacking complete knowledge. Although at the national and international level, research and studies in this field are increasing, it is not always possible to establish how certain diseases occur in wildlife and whether wildlife plays a role in the maintenance and transmission of pathogens [1]. Diseases affecting wild species can occur with different morpho-pathological patterns in a wide range of hosts and populations. Where some occur asymptomatically, with negligible impact on wild populations, domestic animals and humans, they can occasionally cause dramatic epizootic events characterized by high morbidity and mortality [2].

Examples of these events occurring over the past 50 years include North America’s first major outbreak of duck plague that killed more than 40,000 domestic ducks and waterfowl in 1973 [3]; outbreaks of rabbit hemorrhagic disease (RHD) that caused a severe reduction in the wild rabbit populations in the Iberian Peninsula between 1988 and 1989 [4]; a West Nile virus infection that in America in 2003 killed more than 11,000 birds, while in Europe, although it has been reported since the 1950s, caused small outbreaks that were contained [5]. In most cases, the involvement of wildlife in the maintenance of infection is only suspected or hypothesized and is unlikely to be correctly demonstrated. Sometimes, wildlife is attributed responsibilities it does not have, especially when no explanation can be given for the occurrence of diseases in domestic animals.

Clearly defining the epidemiological role of wild animals is particularly important with regard to the World Organization for Animal Health (OIE) List A diseases and, particularly those subject to state public health measures and the zoonotic diseases [6]. For these purposes, the difficulties in the collection of samples to conduct research on wildlife diseases are a significant limitation. The real role of wildlife in the epidemiology of infectious diseases and its interaction with domestic animals, in relation to the characteristics of the territory, the types of farming and the management of the fauna, still deserve in-depth knowledge.

It is well known and widely reported that wild animals play a role in the maintenance of pathogens, bacteria, and parasites in a territory, some of which are responsible for zoonoses [7]. Cattle, sheep, and goat farms frequently use state parks and marginal areas as a useful forage resource, sometimes forming an integral part of the local fauna; indeed, it is not uncommon to observe areas where domestic species (cattle, sheep, and goats) and wild species (wild boar, rabbit, and hares) graze simultaneously [8].

Another critical concern regards the possibility that these animals could have a role in the diffusion of antimicrobial resistance (AMR) [9]. Although for decades, the scientific community has promoted the prudent use of antibiotics in an attempt to slow down the spread of resistance, this has not always been applied in contexts, such as agriculture and zootechny. As a result, there has been the dissemination of antimicrobials and resistant bacteria not only through urban sewage but also through the field and livestock wastewater [10]. Therefore, in the natural environment, the spread of antimicrobial resistance is determined by a mixture of factors, including the exposure to antimicrobial drugs that can promote the selection of resistant bacteria, the transmission of genetic determinants favored by the high abundance and density of bacteria in natural environments, and the presence of optimal conditions for the transmission of AMR genes from commensal and environmental to pathogenic bacteria [11,12,13].

The acquisition of resistance genes is considered the main factor that contributes to the wide distribution and diffusion of antimicrobial resistance; indeed, through vertical or horizontal transfer, there is an exchange of elements, such as plasmids and transposons. Transported by plasmids or contained within transposons, there are integrons that provide bacteria with a gene capture system perfectly adapted to the challenges of multiple antibiotic treatment regimens and that have been considered to contribute to the release of “superbugs” [14]. Several studies conducted over the last decade showed that wildlife and, particularly, migratory birds could acquire resistant bacteria present in contaminated environments and become a reservoir and carrier for AMR spread [15,16,17,18].

Based on these observations, the presence of pathogenic bacteria, including zoonotic agents, in animals (mammals, reptiles, and birds) from different areas of Sicily, Italy, was evaluated to monitor the health status of wildlife populations. To better understand the spread of antimicrobial resistance in wild animals as part of a One Health approach, the susceptibility profile of some of the isolated bacterial strains was determined by microbiological assays, and the presence of genes coding for antimicrobial resistance was evaluated by molecular analysis.

## 2. Materials and Methods

### 2.1. Sample Collection

**Figure 1:**
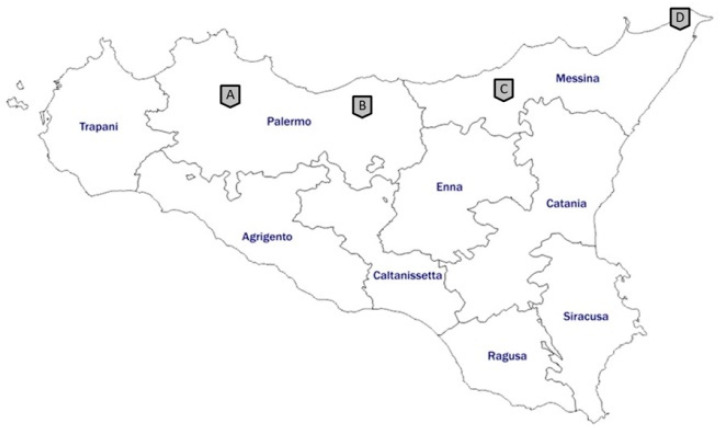
Map of Sicily showing where the animals were obtained. Site A: Wildlife Rescue Center of Bosco di Ficuzza; site B: Parco delle Madonie; site C: Parco dei Nebrodi; site D: Wildlife Rescue Center “Stretto di Messina”.

Between 2017 and 2019, samples from 193 wild birds, 119 mammals, 35 terrestrial, and 21 swamp tortoises, from occasionally found carcasses, hunting activities, and regional recovery centers, located mainly in the provinces of Messina and Palermo, were analyzed (Figure 1).

The recovery centers collect wounded live animals that need care and carcasses from most of the Sicilian territory, although they are located near two of the largest Sicilian regional parks (Parco delle Madonie and Parco dei Nebrodi). Before sample collection, each animal was identified on a morphological basis to define the species. The 275 carcasses were subjected to necropsy, including gross examinations and tissue sample collections from the organs (intestines, liver, spleen, kidneys, heart, and lungs) for laboratory investigations.

From the live animals, mainly birds and turtles, housed in recovery centers, samples, including 79 swabs (oral, skin, rectal/cloacal, tracheal, of lesions) and 14 feces, were collected. These samples were collected by veterinary practitioners, who visited the rescued animals in the recovery centers, and were sent to the laboratory to facilitate the diagnosis and perform appropriate antibiotic treatments prior to their release into the wild. In addition, feces samples and rectal/cloacal swabs were screened for pathogenic bacteria that were potentially transmissible to other animals housed in the centers.

### 2.2. Bacterial Isolation and Identification

Swabs and feces from live animals and tissue samples collected from carcasses were subjected to bacteriological procedures to isolate bacterial species. Bacteriological cultures were performed using selective and differential medium (blood agar, MacConkey agar, and mannitol salt agar, Thermo Fisher Scientific, Waltham, MA USA) to promote the growth of the main commensal and pathogenic species from the collected samples [19,20]. For the isolation of *Salmonella* spp. species, a pre-enrichment in buffered peptone water (APT) and two subsequent enrichments in selenite cystine and Rappaport–Vassiliadis broths (Thermo Fisher Scientific, MA USA), incubated, respectively, at 37 °C and 42 °C for 24 h, were performed from the intestine, spleen, liver, feces, and rectal/cloacal swabs. The enrichment broths were then seeded in xylose lysine desoxycholate (XLD) and brilliant green agar (BGA) [6]. The colonies isolated on the different agars were identified using biochemical-enzymatic tests.

For the most significant strains, amplification and nucleotide sequencing of the 16S rRNA genes was performed. Bacterial DNA was extracted using 100 µL of PrepMan™ ultra sample preparation reagent (Thermo Fisher Scientific, MA USA), according to the protocol indicated by the manufacturer. The Q5^®^ high-fidelity DNA polymerase kit (New England BioLabs, UK) and a pair of universal primers (5′-CCAGCAGCCGGCGGGTAATACG-3′ as the forward primer and 5′-ATCGGYTACCTTGTTACGACTTC-3′ as the reverse primer) were used in a polymerase chain reaction (PCR) assay [21].

An aliquot of extracted DNA (5 µL) was added to a PCR reaction mix prepared by mixing a final concentration of 1X of Q5 reaction buffer, 200 µM of dNTPs, 0.5 µM of each universal primer, 0.02 U/µL of Q5 Hot Start high fidelity DNA polymerase, and 1X of Q5 High GC enhancer, in a total volume of 50 µL. After 10 min of denaturation at 98 °C, the reaction mixture was run through 35 cycles of denaturation for 30 s at 98 °C, annealing for 1 min at 55 °C, and extension for 30 s at 72 °C, followed by a final extension of 2 min at 72 °C. Subsequently, 10 µL of the PCR product was used for electrophoresis on a 1% agarose gel to determine the size of the product. The polymerase chain reaction (PCR) products were purified and sequenced at BMR Genomics Srl (Padova, Italy). The nucleotide sequences were identified using the National Center for Biotechnology Information (NCBI, Bethesda, MD, USA) nucleotide basic local alignment search tool (BLASTn) program.

Unfortunately, only 61 strains of the 222 isolates at the first stage were successfully recovered in purity after storage. However, the analyzed strains were representative of the species present in the tested animals.

### 2.3. Antimicrobial Susceptibility Using the Disk Diffusion Method

The antibiotic susceptibility of 61 isolated strains was determined by the disk diffusion method on Mueller–Hinton agar (Thermo Fisher Scientific, MA, USA) (Kirby–Bauer). A set of 12 antibiotics representative of the main classes used in human and veterinary medicine was chosen: amikacin (30 µg), gentamicin (10 µg), ampicillin (10 µg), amoxicillin/clavulanic acid (30 µg), ceftiofur (30 µg), ceftriaxone (30 µg), clindamycin (2 µg), chloramphenicol (30 µg), ciprofloxacin (5 µg), enrofloxacin (5 µg), sulfamethoxazole/trimethoprim (23.75 + 1.25 µg), and tetracycline (30 µg). The interpretation of the results was performed in accordance with the Clinical and Laboratory Standards Institute (CLSI) ranges [22]. However, the CLSI ranges for some of the tested molecules were not available, and the European Committee on Antimicrobial Susceptibility Testing (EUCAST) ranges were used [23].

### 2.4. Detection of Antibiotic Resistance Genes and Class-1 Integron

The presence of *bla_TEM_*, *bla_CTXM_*, *tetA*, *qnrS,* and *sull II* antibiotic resistance genes and of the mobile element *int1* in 61 bacterial strains was determined using PCR. These genes were chosen for their contribution to resistance against the most commonly used antibiotics, especially in veterinary medicine, and to assess any correspondence between the phenotypic resistance found and the presence of these genes in the tested strains. A PCR reaction mix containing 5 µL of the DNA template and a final concentration of 1X DreamTaq buffer, 2 mM of dNTPs, 0.5 μM of the forward primer, 0.5 μM of the reverse primer, and 1.25 U of DreamTaq DNA polymerase (Thermo Fisher Scientific, Waltham, MA USA) in a total volume of 50 µL was utilized. Subsequently, 10 µL of the PCR product was used for electrophoresis on a 1% agarose gel to determine the size of the product. The primers and the annealing temperatures used are reported in Table 1.

## 3. Results

### 3.1. Host Animal Species and Samples Collected

During the three-year period 2017–2019, 275 animal carcasses, 79 fecal samples, and 14 swabs from 368 wild animals were analyzed. Details on the species and number of the animals tested are presented in Table 2. Most of the species analyzed belonged to the class of birds (*n* = 193), followed by mammals (*n* = 119), and, with a minor number, reptiles (*n* = 56).

All carcasses were subjected to autopsy, which revealed several anatomopathological lesions, many of which can be attributable to direct contact with humans. For the birds, indeed, the most frequent causa mortis was from gunshot wounds with fractured forelimbs; moreover, many of the subjects analyzed appeared emaciated and dehydrated. During the autopsy of mammals, subcutaneous hematomas, abdominal hemorrhagic effusions with a ruptured spleen, and fractures were observed, all injuries attributable to impact trauma. Thus, fecal samples and swabs (oral, skin, rectal/cloacal, tracheal, and lesions) from live animals were submitted to the laboratory by veterinary practitioners of the recovery centers who suspected bacterial infections, which were then confirmed by laboratory analyses and subsequently treated during the hospitalization before the release of the animal in the wild.

### 3.2. Bacterial Detection

The bacteriological examination conducted on different samples from 368 wild animals showed the presence of one or more bacterial species in 60.4% (222/368) of the subjects analyzed. The most isolated genus was *Escherichia,* found in 114 animals (51.3%), followed by *Clostridium* (22.5%), *Citrobacter* (16.6%), and *Aeromonas* (14.4%) (Table 3). The most isolated species were *Escherichia coli* (104/222, 46.84%), *Clostridium perfringens* (50/222, 25.5%), and *Citrobacter freundii* (29/222, 13.06%). Unique bacteria species were isolated in 57.6% (128/222) of the subjects, whereas in 14.4% (32/222) and 24.7% (55/222) of the subjects, two and three bacteria species were isolated, respectively. In addition, the presence of four different bacterial species was found in six animals, while five were isolated in only one subject. The prevalence of the isolated bacterial genera varied among the host classes (Table 3).

### 3.3. Antimicrobial Susceptibility Using the Disk Diffusion Method

The susceptibility to the chosen set of antibiotics was determined for 61 strains. The results of the antibiograms performed with the Kirby–Bauer method are reported in Table 4. The highest resistance values were found against ampicillin (41.8%), excluding *Enterobacterales* species, such as *Citrobacter* spp. and *Klebsiella* spp., whose resistance to this antibiotic is indicated as intrinsic by EUCAST. This resistance was present, in particular among strains isolated from mammals (7/12, 58.3%) and birds (22/41, 53.7%). The results obtained for the other antibiotics tested showed high percentages of susceptibility, particularly to chloramphenicol (93%), ceftriaxone (90%), sulfamethoxazole/trimethoprim (85%), and amoxicillin/clavulanic acid (84%).

In addition, the results obtained showed the presence of seven multidrug-resistant strains (MDR), five of which were resistant to three antibiotic classes, one to four, and another to five. Specifically, the MDR strains were: two *E. coli* from one owl (id. 13) and one tortoise (id. 10) resistant to penicillins, sulfonamides, and tetracyclines; *E. coli* from one kestrel (id. 28) resistant to penicillins, cephalosporins, and aminoglycosides; *Campylobacter jejuni* from a crow (id. 30) resistant to penicillins, cephalosporins, and sulfonamides; *Pseudomonas aeruginosa* from a peregrine falcon (id. 33) resistant to cephalosporins, lincosamides, and sulfonamides; *Enterobacter cloacae* from a crow (id. 25) resistant to penicillins, cephalosporins, sulfonamides, and tetracyclines; and *Staphylococcus simulans* from a peregrine falcon (id. 32) resistant to penicillins, phenols, fluoroquinolones, lincosamides, and tetracyclines.

### 3.4. Detention of Resistance Genes and Int1

Molecular analysis conducted for the detection of resistance genes (*bla_TEM_, bla_CTXM_, tetA, sulII,* and *qnrS*) and mobile element class-1 integron (*int1*) demonstrated a reduced incidence of these in the bacterial strains isolated. Indeed, of the 61 strains analyzed, only seven (11.47%) were positive for the presence of one or more genes among those investigated. These seven strains, all belonging to the *Enterobacterales* family, were three strains of *E. coli* (from one tortoise id. 10 and two peregrine falcons id. 15 and 33) in which only the *bla_TEM_* gene was present, an *E. coli* (from golden eagle id. 5), which harbored two resistance genes, *bla_TEM_* and *sulII*, and *E. cloacae* (from rabbit id. 25), in which *tetA* and *sulII* were present.

Three resistance genes, *bla_TEM_*, *tetA,* and *sulII*, were found in two strains of *E. coli* (from owl id. 13 and flamingo id. 26). The presence of *bla_CTXM_* and *qnrS* genes was not found in any of the strains tested. Finally, we found the presence of the mobile element *int1* in three strains (5.2%), two *E. coli,* from golden eagle id. 5 and owl id. 13 and *E. cloacae* from rabbit id. 25. Table 5 reports the results of the antibiograms and resistance genes from the seven strains that tested positive for one or more of the genes.

## 4. Discussion

This study reports the data regarding the presence of antimicrobial resistance in bacteria isolated in 2017–2019 from wildlife in Sicily. Most of the analyzed samples were collected from subjects with death due to anthropic activities (gunshot wounds or impacts with vehicles). Bacteriological analysis allowed the isolation of different bacterial species. Although some of the bacterial species isolated may have pathogenic and/or zoonotic potential, in most cases, they were bacterial species commensal with those animals, without pathogenic activity, and were not related to the anatomopathological findings observed during post-mortem inspection. Only in a few cases, for example, in live animals with wounds or skin lesions infected by *Staphylococcus aureus* or *P. aeruginosa*, were the isolated bacteria responsible for the clinical signs of the lesions.

In fact, although wildlife could represent a risk to humans and domestic animals when it acts as a reservoir of disease, intermediate host, or biological amplifier, most interactions between microorganisms and wildlife are harmless and present relatively few risks [1]. The analyses performed indicated that the microorganisms belonging to the family *Enterobacterales* (76%) represent the species most prevalent in wildlife. This result is in accordance with those reported in previous studies on wild birds in Sicily, in which the most commonly reported species were *E. coli*, *C. freundii,* and *Klebsiella oxytoca* [25]. In addition, *C. perfringens*, which, after *E. coli,* was the most isolated species from both birds and mammals, was indicated as a possible part of the microbiota in wild animals by some authors, although more studies on its role in the wild are needed [26,27].

The phenotypic resistance was evaluated by testing antibiotics belonging to eight different classes: penicillin (ampicillin and amoxicillin/clavulanic acid), cephalosporins (ceftriaxone and ceftiofur), phenicols (chloramphenicol), fluoroquinolones (enrofloxacin and ciprofloxacin), aminoglycosides (gentamicin and amikacin), lincosamides (clindamycin), tetracyclines (tetracycline), and sulfonamides (sulfamethoxazole/trimethoprim). To avoid overestimating the incidence of resistance, the data obtained were interpreted, taking into account, in accordance with the bacterial species tested, the intrinsic resistance reported by EUCAST [24].

The data we collected showed the percentage of resistance to the antibiotics tested as less than or equal to 16%, except for ampicillin, for which the detected resistance was 41.8%. However, in a previous study conducted on Gram-negative bacteria isolated from wild birds in Sicily in 2013, significant levels of resistance against sulfamethoxazole/trimethoprim and amoxicillin/clavulanic acid were reported in a population of live wild birds [25]. High percentages of resistance in *Enterobacterales* isolated from wild animals were also found in other Italian regions: strains resistant to cephalothin (94.3%), amoxicillin/clavulanic acid (86.9%), and tetracycline (44.6%) were found in wild boars in Tuscany, while, in strains from migratory Passeriformes transiting through the Metaponto territory (Basilicata, Italy), resistances to amoxicillin (64. 8%), ampicillin (63.1%), rifampicin (61.5%), and amoxicillin-clavulanic acid (54.1%) were found [28,29].

In addition, in contrast to our data, a high incidence of resistance to several antibiotics was also reported in studies conducted on wild species from other European areas: Wasyl et al. detected resistance to 11 antibiotic molecules, including streptomycin, tetracycline, sulfamethoxazole/ trimethoprim, and colistin, in *E. coli* isolated from wild boar and deer in Poland; Smith et al. in a study conducted on herring gulls and deer in Ireland found that all strains isolated were resistant to rifampicin, oxacillin, and penicillin; and a moderately high level of resistance to tetracycline, streptomycin, ampicillin, and sulfamethoxazole/trimethoprim (35%) was found in fecal samples obtained from wild animals in Portugal [30,31,32].

The results in the present study showed that, except for a multidrug-resistant strain from a terrestrial turtle, the other six MDR strains were isolated from wild birds (Table 4), as well as the majority of strains carrying at least one resistant gene (Table 5). These may be explained by the fact that birds come in contact with many different environments, dry and wet areas, and water resources close to human settings. Predatory birds can also feed on carcasses of livestock animals that can carry more AMR strains.

To confirm these data, further sampling, including more subjects from the different classes of animals, will be necessary. However, the data collected regarding the antibiotic susceptibility, determined using the phenotypic method, indicated that resistance at least for the 12 antibiotics tested in this study was not widespread among strains isolated from the wild animals in our study. In contrast to data related to companion animals, which showed a higher prevalence of resistant bacteria [33], the results of this study indicate that, in wildlife, antimicrobial resistance may be quite unusual and that the major responsibility for high resistance is related to the human factor.

A low incidence of genes for antimicrobial resistance was also present in these strains from the wild. Although they were investigated in both Gram-positive and negative strains, these genes have only been found in a few strains (11.4%, 7/61) belonging to the *Enterobacterales* family, and mainly in *E. coli* strains (6/7). All these *E. coli* harbored the *bla_TEM_* genes, which, together with the *bla_CTXM_* absent in our strains, are the most widespread resistance genes. The *bla_TEM_* gene coding for β-lactamase TEM is the most common mechanism of resistance to ampicillin in *E. coli*.

Although 87% (20/23) of the *E. coli* strains tested were found to be phenotypically resistant to ampicillin, only for six of these strains, could the resistance be attributable to the presence of resistance genes. In fact, as reported by several authors, there are multiple complex mechanisms that lead a bacterium to be resistant to antimicrobials, and phenotypic expression does not always correspond to genotypic resistance and vice versa [18,34,35]. This is evident in *E. coli* strains id. 15, 33, and 10, which, although they harbored only the *bla_TEM_* gene, had different phenotypic resistance profiles. Indeed, while strain id. 15 was resistant only to ampicillin, the other two strains were also resistant to other antibiotics: strain id. 33 showed resistance also to amikacin and strain id 10, also to sulfamethoxazole/trimethoprim and tetracycline (Table 5).

For the same reason, *sulII (*responsible for resistance to sulfonamides) and *tetA* (to tetracyclines) were found in only four (three *E. coli* and one *E. cloacae*) and three strains (two *E. coli* and one *E. cloacae*), respectively, although not all these strains showed phenotypic resistance to these antibiotics.

Three of the strains that harbored resistance genes were also found to contain class-1 integron, a mobile element carrying resistance cassettes that has been identified in Gram-negative species, mainly *E. coli*, and some Gram-positive species, such as *Staphylococcus* spp. and *Corynebacterium* spp. of clinical origins [36,37]. With regard to the presence of the class-1 integron in wild animals, a study conducted on different animal populations subject to different types of anthropogenic interference demonstrated that its abundance varied depending on the proximity to humans [38]. In addition, class 1 integrons can be spread through urban, agricultural, and livestock wastewater, and, through wastewater treatment plants, they can reach aquatic environments, such as the rivers and estuaries often used by wildlife [39,40]. However, the reduced presence of class-1 integrons (6.5%) detected in our study could be explained by the fact that the genome of bacteria, such as *E. coli,* is not able to acquire or maintain class-1 integrons without antibiotic pressure [41] and that, also considering the results of phenotypic resistance, in the Sicilian wild environment, the antibiotic pressure does not appear to be high.

## 5. Conclusions

The natural microbiota of animals, as well as humans, are subject to numerous antimicrobial pressures due not only to the use and abuse of antibiotic drugs but also to their use in agriculture. In addition, antibiotics and resistant bacteria present in urban, agricultural, and livestock wastewater can also arrive in wild environments where factors, such as the huge number of bacterial species present and the high-density, can promote gene exchange between bacteria and the spread of antibiotic resistance.

Although this study demonstrated a reduced prevalence of pathogenic and/or zoonotic bacteria, as well as a reduced prevalence of phenotypic resistance and genes determining resistance to the main antibiotic classes, in a One Health approach, it is particularly important to monitor wildlife. One Health is an ideal approach to achieve global health by recognizing that human, animal, and ecosystem health are inextricably linked. In fact, antimicrobial resistance is now considered a zoonotic health threat. Worldwide, antimicrobial resistance is on the rise, and, if current trends continue unabated, it has been estimated that this could be the cause of 10 million deaths by 2050 [42].

Wild animals, especially species such as wild boars, foxes, and seagulls, which are increasingly closer to human environments, can be reservoirs and diffusers not only of pathogenic and/or zoonotic bacteria but also of resistant commensal bacteria or bacteria that possess potentially transferable antibiotic resistance genes. Even in aquatic environments, including remote ones, such as glaciers, the presence of antimicrobial-resistant bacteria has been detected [43]. Thus, in addition to the guidelines on antibiotic use in humans and animals, a more comprehensive approach is needed, including constant monitoring of wildlife, aquaculture, and the environment to assess and control the spread of bacteria and the determinants of antimicrobial resistance.

## Figures and Tables

**Table 1:** Primers used in this study.

Target	Primer Sequence (5′–3′)	Annealing Temperature (°C)	Amplicon Size (bp)
*tetA*	GCTACATCCTGCTTGCCTTC CATAGATCGCCGTGAAGAGG	58	210
*qnrS*	GACGTGCTAACTTGCGTGAT TGGCATTGTTGGAAACTTG	60	118
*sull II*	TCCGGTGGAGGCCGGTATCTGG CGGGAATGCCATCTGCCTTGAG	60	191
*bla_TEM_*	TTCCTGTTTTTGCTCACCCAG CTCAAGGATCTTACCGCTGTTG	58	112
*bla_CTXM_*	CTATGGCACCACCAACGATA ACGGCTTTCTGCCTTAGGTT	58	103
*int1*	GGCTTCGTGATGCCTGCTT CATTCCTGGCCGTGGTTCT	55	148

**Table 2:** The wild animal species analyzed.

Class	Common Name (*Scientific Name*)	Number
Bird	Gray Heron (*Ardea cinerea*)	2
	Tawny owl (*Strix aluco*)	10
	European scops owl (*Otus scops*)	1
	Golden eagle (*Aquila chrysaetos*)	2
	Barn Owl (*Tyto alba*)	10
	Egyptian vulture (*Neophron percnopterus*)	1
	White stork (*Ciconia ciconia*)	2
	Great cormorant (*Phalacrocorax carbo*)	1
	Hooded crow (*Corvus cornix*)	1
	Crow (*Corvus corax*)	1
	Lanner Falcon (*Falco biarmicus*)	3
	Peregrine Falcon (*Falco peregrinus*)	10
	Honey Buzzard (*Pernis apivorus*)	13
	Flamingo (*Phoenicopterus ruber*)	6
	Seagull (*Larus ridibundus*)	15
	Royal Seagull (*Larus michahellis*)	20
	Little Egret (*Egretta garzetta*)	2
	Magpie (*Pica piza*)	2
	Kestrel (*Falco tinnunculus*)	27
	Jay (*Garrulus glandarius*)	2
	Gryphon (*Gyps fulvus*)	1
	Bee-eater (*Merops apiaster*)	1
	Owl (*Asio otus*)	1
	Royal Kite (*Milvus milvus*)	1
	Buzzard (*Buteo buteo*)	52
	Squacco Heron (*Ardeola ralloides*)	1
	Great Crested Grebe (*Podiceps cristatus*)	2
	Little bittern (*Ixobrychus minutus*)	1
	Mallard (*Anas platyrhynchos*)	1
Mammals	Wild boar (*Sus scrofa*)	23
	Rabbit (*Oryctolagus cuniculus*)	84
	Deer (*Dama dama*)	1
	Porcupine (*Hystrix cristata*)	2
	Marten (*Martes martes*)	1
	Hedgehog (*Erinaceus europaeus*)	4
	Fox (*Vulpes vulpes*)	3
	Bat (*Tadarita teniodis*)	1
	Mallard (*Anas platyrhynchos*)	1
Reptiles	Hermann’s tortoise (*Testudo Hermanni*)	29
	Greek tortoise (*Testudo graeca*)	4
	Marginated tortoise (*Testudo marginata*)	2
	Pond slider (*Trachemys scripta*)	19
	Sicilian Pond Turtle (*Emys trinacris*)	2

**Table 3:** The results of the bacterial isolation and prevalence in birds (*n* = 193), mammals (*n* = 119), and reptiles (*n* = 56).

Bacterial Genera	Number of Isolated Bacteria	Prevalence (%)		
		Birds	Mammals	Reptiles
*Escherichia* spp.	114	23.3	52.9	10.7
*Clostridium* spp.	50	14	18.5	1.8
*Citrobacter* spp.	37	0.5	1.7	60.7
*Aeromonas* spp.	32	9.3	5.9	12.5
*Staphylococcus* spp.	27	11.4	1.7	5.4
*Pasteurella* spp.	15		12.6	
*Streptococcus* spp.	12	3.1	4.2	1.8
*Enterobacter* spp.	8	2.1	3.4	
*Klebsiella* spp.	6	2.1		3.6
*Pseudomonas* spp.	5	2.1	0.8	
*Salmonella* spp.	4	0.5		5.4
*Campylobacter* spp.	3	0.5		3.6

**Table 4:** The antibiotic susceptibility results (*n* = 61).

Animal Species	Id	Bacterial Species	AMP	AMC	FUR	CRO	C	ENR	CIP	CN	AK	DA	SXT	TE
Birds (26 individuals)														
Mallard	1	*Escherichia coli*	R	S	R	I	S	S	S	S	S	R ^a^	S	I
Peregrine falcon	4	*Staphylococcus aureus*	R	S	S	S	S	R	R	S	S	S	S	S
		*Escherichia fergusonii*	R	S	R	S	S	S	S	S	S	R ^a^	S	I
Golden Eagle	5	*Staphylococcus aureus*	R	S	S	S	S	R	R	S	S	S	S	S
		*Escherichia coli*	R	S	S	S	S	R	R	S	S	S ^b^	S	S
Kestrel	6	*Escherichia coli*	R	S	S	S	S	S	S	S	S	R ^a^	S	S
Honey Buzzard	7	*Escherichia coli*	R	S	S	S	S	S	S	S	S	R ^a^	S	S
Buzzard	8	*Staphylococcus* *chromogenes*	S	S	S	S	S	S	S	S	S	I	S	S
European scops owl	12	*Escherichia coli*	R	S	S	S	S	S	S	S	I	R ^a^	S	S
Owl	13	*Yersinia nurmii*	R	S	S	S	S	S	S	S	S	R ^a^	S	S
		*Escherichia coli*	R	S	S	S	S	S	S	S	I	R ^a^	R	R
Peregrine falcon	15	*Escherichia coli*	R	I	S	S	S	S	S	S	S	R ^a^	S	S
Barn owl	16	*Clostridium perfringens*	S	S	S	S	S	S	S	R	R	S	S	S
Kestrel	17	*Escherichia coli*	R	S	I	S	S	I	I	I	I	R ^a^	S	S
Buzzard	18	*Clostridium perfringens*	S	S	S	S	S	S	S	R	R	S	S	S
		*Escherichia coli*	I	S	I	S	S	S	S	S	S	R ^a^	S	S
Tawny owl	19	*Clostridium perfringens*	S	S	S	S	S	S	S	R	R	S	S	I
		*Hafnia alvei*	I	R	S	S	S	S	S	S	S	R ^a^	S	R
		*Escherichia coli*	R	S	I	S	S	S	S	I	S	R ^a^	S	S
Peregrine falcon	20	*Escherichia coli*	I	R	S	S	S	S	S	S	S	R ^a^	S	S
		*Hafnia alvei*	I	S	S	S	S	S	S	S	S	R ^a^	S	S
		*Klebsiella oxytoca*	R ^a^	R	S	S	S	S	S	S	S	R ^a^	S	S
Kestrel	23	*Clostridium perfringens*	S	S	S	S	S	S	S	R	R	S	S	I
Kestrel	24	*Pseudomonas aeruginosa*	R ^a^	R ^a^	S	I ^b^	I ^b^	R	R	S	S	R ^a^	R	I ^b^
Flamingo	26	*Escherichia coli*	R	S	I	S	S	I	S	S	S	R ^a^	S	R
Kestrel	28	*Escherichia albertii*	R	S	S	S	S	S	S	S	S	R ^a^	S	S
		*Escherichia coli*	R	S	R	S	S	S	S	I	R	R ^a^	S	S
		*Staphylococcus warneri*	S	S	S	S	S	S	S	S	S	S	S	S
Gray Heron	29	*Salmonella enterica*	R	S	I	S	S	S	S	S	S	R ^a^	S	S
Crow	30	*Staphylococcus aureus*	I	S	I	S	S	S	S	S	S	S	S	S
		*Enterococcus faecium*	S	S	R ^a^	I ^b^	S	S	S	S	S	S	R^a^	S
		*Campylobacter jejuni*	R	S	R	I	S	S	S	S	S	S	R	S
Peregrine falcon	32	*Staphylococcus simulans*	R	S	S	S	R	R	I	S	S	R	S	R
Peregrine falcon	33	*Escherichia coli*	R	S	I	S	S	S	S	I	R	R^a^	S	S
		*Staphylococcus* *chromogenes*	S	S	I	S	S	R	I	S	S	I	S	S
		*Pseudomonas aeruginosa*	R ^a^	R ^a^	R	I ^b^	S ^b^	I	I	S	S	R	R	I ^b^
Peregrine falcon	34	*Escherichia fergusonii*	R	S	I	S	S	S	S	I	R	R ^a^	S	S
		*Pseudomonas aeruginosa*	R ^a^	R ^a^	I	I ^b^	S ^b^	I	I	S	S	I ^b^	R	I ^b^
Bee-eater	35	*Escherichia coli*	R	S	I	S	S	S	S	S	I	R ^a^	S	I
Royal Seagull	38	*Streptococcus bovis*	S	S	S	S	S	I	I	I ^b^	R ^a^	I	R	I
Royal Seagull	39	*Escherichia coli*	I	S	I	S	S	S	S	I	S	R ^a^	S	S
Total resistant strains on 41 isolates			22	3	5	0	1	6	3	4	7	2	6	4
Mammals (9 individuals)														
Deer	2	*Escherichia coli*	R	S	S	S	S	S	S	S	S	R ^a^	S	S
Rabbit	3	*Escherichia coli*	R	S	S	S	S	S	S	S	I	R ^a^	S	S
Fox	9	*Klebsiella oxytoca*	R ^a^	S	S	S	S	S	S	S	S	R ^a^	S	S
Rabbit	14	*Escherichia coli*	R	S	I	S	S	S	S	I	R	R ^a^	S	S
Marten	21	*Escherichia coli*	R	S	S	S	S	S	S	S	S	R ^a^	S	S
Rabbit	22	*Escherichia coli*	R	S	I	S	S	S	S	I	I	R ^a^	S	S
Rabbit	25	*Enterobacter cloacae*	R	I	R	S	S	S	S	S	I	R ^a^	R	R
Hedgehog	27	*Citrobacter freundii*	R ^a^	R ^a^	S	S	S	I	S	S	S	R ^a^	S	S
		*Streptococcus dysgalactiae*	S	S	S	S	S	I	I	S ^b^	R ^a^	I	S	S
		*Staphylococcus simulans*	S	S	S	S	S	S	S	S	S	S	S	S
Wild boar	31	*Escherichia fergusonii*	R	S	I	S	S	S	S	S	S	R ^a^	S	S
		*Citrobacter freundii*	R ^a^	S ^b^	I	S	S	S	S	S	S	R ^a^	S	S
Total resistant strains on 12 isolates			7	1	0	0	0	0	0	0	1	0	1	1
Reptiles (4 individuals)														
Herman’s tortoise	10	*Escherichia coli*	R	S	S	S	S	S	S	S	S	R ^a^	R	R
		*Citrobacter braakii*	R ^a^	S ^b^	S	S	S	S	S	S	S	R ^a^	S	S
		*Salmonella tennessee*	R	S	I	S	S	S	S	S	S	R ^a^	S	S
Greek tortoise	11	*Corynebacterium xerosis*	S	S	S	S	S	S	S	S	S	I	S	S
		*Escherichia coli*	R	I	R	S	S	S	S	S	S	R ^a^	S	I
Sicilian Pond Turtle	36	*Providencia rustigianii*	S	S	S	S	S	S	S	S	S	R ^a^	S	R
		*Klebsiella oxytoca*	R ^a^	S	I	S	S	S	S	S	I	R ^a^	S	S
Sicilian Pond Turtle	37	*Klebsiella oxytoca*	R ^a^	S	I	S	S	S	S	S	I	R ^a^	S	S
Total resistant strains on 8 isolates			3	0	2	0	0	0	0	0	0	0	1	2

Ampicillin (AMP); amoxicillin/clavulanic acid (AMC); ceftiofur (FUR); ceftriaxone (CRO); chloramphenicol (C); enrofloxacin (ENR); ciprofloxacin (CIP); gentamycin (CN); amikacin (AK); clindamycin (DA); sulfamethoxazole/trimethoprim (SXT); tetracycline (TE); Resistant (R); susceptible (S); Intermediate (I); ^a^ Reported as intrinsic resistance [24]; ^b^ Reported as intrinsic resistance in [24].

**Table 5:** Resistance genes and antibiograms with the Kirby–Bauer method (*n* = 7).

Animal Species	Id	Bacterial Species	Resistance Genes	AMP	AMC	FUR	CRO	C	ENR	CIP	CN	AK	DA	SXT	TE
Golden Eagle	5	*Escherichia coli*	*bla_TEM_, sullII, int1*	R	S	S	S	S	R	R	S	S	S ^b^	S	S
Owl	13	*Escherichia coli*	*tetA, bla_TEM_, sullII, int1*	R	S	S	S	S	S	S	S	I	R ^a^	R	R
Peregrine falcon	15	*Escherichia coli*	*bla_TEM_*	R	I	S	S	S	S	S	S	S	R ^a^	S	S
Flamingo	26	*Escherichia coli*	*tetA, bla_TEM_, sullII*	R	S	I	S	S	I	S	S	S	R ^a^	S	R
Peregrine falcon	33	*Escherichia coli*	*bla_TEM_*	R	S	I	S	S	S	S	I	R	R ^a^	S	S
Rabbit	25	*Enterobacter cloacae*	*tetA, sullII, int1*	R	I	R	S	S	S	S	S	I	R ^a^	R	R
Herman’s tortoise	10	*Escherichia coli*	*bla_TEM_*	R	S	S	S	S	S	S	S	S	R ^a^	R	R

ampicillin (AMP); amoxicillin/clavulanic acid (AMC); ceftiofur (FUR); ceftriaxone (CRO); chloramphenicol (C); enrofloxacin (ENR); ciprofloxacin (CIP); gentamycin (CN); amikacin (AK); clindamycin (DA); sulfamethoxazole/trimethoprim (SXT); tetracycline (TE); Resistant (R); susceptible (S); Intermediate (I); ^a^ reported as intrinsic resistance [24]; ^b^ reported as intrinsic resistance in [24].

## Data Availability

All data discussed are contained in the article. For 16S rRNA sequences, please contact the corresponding author.
